# Tetracyclines in malaria

**DOI:** 10.1186/s12936-015-0980-0

**Published:** 2015-11-10

**Authors:** Tiphaine Gaillard, Marylin Madamet, Bruno Pradines

**Affiliations:** Unité de Parasitologie, Département d’Infectiologie de Terrain, Institut de Recherche Biomédicale des Armées, Marseille, France; Unité de Recherche sur les Maladies Infectieuses et Tropicales Emergentes, UM 63, CNRS 7278, IRD 198, Inserm 1095, Aix Marseille Université, Marseille, France; Fédération des Laboratoires, Hôpital d’Instruction des Armées Saint Anne, Toulon, France; Equipe Résidente de Recherche en Infectiologie Tropicale, Institut de Recherche Biomédicale des Armées, Hôpital d’Instruction des Armées, Marseille, France; Centre National de Référence du Paludisme, Marseille, France; Unité de Parasitologie et d’Entomologie, Département des Maladies Infectieuses, Institut de Recherche Biomédicale des Armées, Brétigny sur Orge, France

**Keywords:** Malaria, *Plasmodium falciparum*, Anti-malarial drug, Resistance, Tetracycline, Doxycycline, Prophylaxis, Treatment

## Abstract

Malaria, a parasite vector-borne disease, is one of the greatest health threats in tropical regions, despite the availability of malaria chemoprophylaxis. The emergence and rapid extension of *Plasmodium falciparum* resistance to various anti-malarial drugs has gradually limited the number of potential malaria therapeutics available to clinicians. In this context, doxycycline, a synthetically derived tetracycline, constitutes an interesting alternative for malaria treatment and prophylaxis. Doxycycline is a slow-acting blood schizontocidal agent that is highly effective at preventing malaria. In areas with chloroquine and multidrug-resistant *P. falciparum* parasites, doxycycline has already been successfully used in combination with quinine to treat malaria, and it has been proven to be effective and well-tolerated. Although not recommended for pregnant women and children younger than 8 years of age, severe adverse effects are rarely reported. In addition, resistance to doxycycline is rarely described. Prophylactic and clinical failures of doxycycline have been associated with both inadequate doses and poor patient compliance. The effects of tetracyclines on parasites are not completely understood. A better comprehension of the mechanisms underlying drug resistance would facilitate the identification of molecular markers of resistance to predict and survey the emergence of resistance.

## Background

Malaria, a parasite vector-borne disease, is one of the greatest health threats in tropical regions, despite the availability of malaria chemoprophylaxis and the use of repellents and insecticide-treated nets. Malaria prophylaxis and chemotherapy remain a major focus of research, and new molecules are constantly being developed prior to the emergence of drug-resistant strains of the malaria parasite. The use of anti-malarial drugs is conditioned on the resistance level of *Plasmodium falciparum* in endemic areas, as well as the contraindications, clinical tolerance and financial costs of these drugs. Among the compounds potentially used against *Plasmodium*, antibiotics have been examined in vitro or in vivo.

Tetracyclines, a family of broad-spectrum antibiotics discovered in the early 1940s, are active in protozoa, including *Plasmodium*. In a small series of patients in 1950, tetracyclines were used to treat *P. falciparum* and *Plasmodium vivax* uncomplicated malaria. The emergence of chloroquine resistance in the 1960s led to studies conducted by the Centers for Disease Control and Prevention (CDC) and the development of the World Health Organization (WHO) recommendations that were based on the use of doxycycline for chemoprophylaxis of falciparum malaria in 1985. Currently, doxycycline is used in combination with quinine in treatment therapies and for chemoprophylaxis in multidrug resistance areas, particularly Southeast Asia. Finally, many armies use it as first-line chemoprophylaxis in areas with chloroquine resistance, including French military forces deployed in malaria-endemic areas. Since 2002, the French Army has, regrettably, had 3000 malaria cases. Recent deployments in Mali and Central African Republic showed high incidence rates, with a significant risk of contracting malaria for the 2000 soldiers. The attack rates were estimated at 7.5 % in 2013 and 12.5 % in 2014. These failures of prophylaxis with doxycycline are mainly associated with inadequate dosing or poor compliance. The pharmacokinetics of doxycycline, including a reduced half-life, may partly explain these failures; however, resistance phenomena may also be a factor.

## Classification

Tetracyclines are synthetic antibiotics derived from a cycline that is naturally produced by bacteria from the genus *Streptomyces* [1]. Tetracycline consists of three groups, based on pharmacological differences: the long-acting group, which includes doxycycline and minocycline, are the most active against *Plasmodium* in vitro. The antibiotic action, common to all tetracycline, is bacteriostatic and inhibits bacterial protein synthesis; their spectrum of activity is large [2].

## Pharmacological properties

The pharmacokinetics properties of doxycycline have been investigated in numerous studies with healthy volunteers. One important property of doxycycline is its ability to be rapidly absorbed orally; it is detectable in the blood 15–30 min after its administration [3, 4]. After an oral dose of 200 mg, peak plasma levels are obtained in approximately 2 h; its half-life ranges from 15 to 25 h [5]. There are great individual variations, depending on the age of the patient and any coadministered substances [6]. Only one study of the pharmacokinetics of doxycycline was conducted during infections. It involved a case of uncomplicated malaria in combination with quinine or artesunate [7]. The authors concluded that there was a need for an initial dose of 400 mg twice daily to maintain plasma concentrations at therapeutic levels during the treatment for malaria infection.

## Side effects and warnings against doxycycline

Tetracyclines are well known for their use in treating bacterial infections, and their adverse effects have been well documented [8, 9]. At the usual doses prescribed for malaria chemoprophylaxis, the published data are limited, and the reported adverse events vary widely. Comparative studies of the tolerance of doxycycline have been contradictory. Several retrospective studies of military teams have reported increased digestive and skin disorders and headaches with chemoprophylaxis [10–14]. A detailed analysis of studies reporting high numbers of side effects makes it possible to objectify pitfalls in the data interpretation: the dosage form is rarely specified and doxycycline is often co-administered with other substances, such as quinine. Thus, it is difficult to attribute an adverse event to cyclines only. In 1996 in sub-Saharan Africa, the French Army Health Service conducted an efficacy study of doxycycline hyclate salt versus chloroquine-proguanil [15].

Doxycycline hyclate was more efficacious than chloroquine-proguanil. However, with a 6 % withdrawal rate due to gastrointestinal side effects, it was considered to be unacceptable as chemoprophylaxis. The gastrointestinal side effects (e.g., diarrhoea and epigastralgia) were attributed to the hyclate salt acidity (pH 3) and the galenic form (capsule). According to the French Drug Agency recommendations, doxycycline hyclate has been replaced by doxycycline monohydrate, a less acidic salt (pH 6) with the same bioavailability [16]. Gastrointestinal side effects, mouth ulcers, and sun sensitization occurred less frequently in the doxycycline monohydrate group than in the chloroquine-proguanil group [17]. Fifty-seven per cent of deployed Australian soldiers using mefloquine prophylaxis in East Timor reported at least one adverse effect, compared to 56 % using doxycycline [18]. In Turkish troops deployed in Afghanistan, the total number of side effects in the doxycycline group was significantly higher than that in the mefloquine group [19]. However, among non-immune travellers to Sub-Saharan Africa, the total number of side effects in the doxycycline group was significantly lower compared with the chloroquine-proguanil or mefloquine groups [20].

The use of an antibiotic for several months for prophylaxis always triggers opposition from a number of bacteriologists, who note the risk of selecting resistant bacteria cyclines [21]. In 1988, a publication reported tetracycline-resistant cases of *Campylobacter jejuni* gastroenteritis among American soldiers based in Thailand [22]. A subsequent study by the same team showed that taking doxycycline for malaria prophylaxis resulted in less exposure to resistant bacteria than the acquisition of already resistant bacteria cyclines, which has long been widespread in this country [23]. The increase in multidrug-resistant gram-negative bacteria colonization among US military personnel in Afghanistan is likely due to environmental exposures rather than doxycycline exposure [24]. Methicillin-susceptible *Staphylococcus aureus* and methicillin-resistant *Staphylococcus aureus* colonization of military personnel under deployment was not associated with doxycycline exposure [25]. However, outbreaks of Panton-Valentine leukocidin-positive, doxycycline resistant, methicillin-susceptible *Staphylococcus aureus* infections associated with doxycycline prophylaxis have been reported in the French Army at the Ivory Coast [26]. Except for these military clinical cases, no study has been published about the risk of bacterial resistance to tetracyclines associated with their prophylaxis use. Doxycycline is contraindicated in patients with allergies to tetracyclines, pregnant women (from the second trimester of pregnancy due to the risk of abnormal tooth bud) and children under 8 years of age because of the risk of discolouration and enamel hypoplasia.

## Mechanism of action

Cyclines are a family of antibiotics that act by inhibiting bacterial protein synthesis. Their mechanisms of action have been described at the molecular level [27]. Cyclines act by binding to several proteins in the 30S ribosomal small subunit and to different ribonucleic acids in the 16S ribosomal RNA. Their mechanisms of action on *Plasmodium* have not been as well described, although a number of studies have addressed this issue. There are three categories of ribosomes in *Plasmodium*: mitochondrial, plastid and nuclear [28]. As suggested by three studies [29–31], tetracycline may directly inhibit mitochondrial protein synthesis and also decrease the activity of a mitochondrial enzyme (i.e., dihydroorotate dehydrogenase) involved in de novo pyrimidine synthesis [32]. Doxycycline inhibits the synthesis of nucleotides and deoxynucleotides in *P. falciparum* [33], but the concentration used (200 µM) is much higher than that used clinically. *In vitro* exposure of *P. falciparum* to minocycline also decreases the transcription of mitochondrial genes (subunit I of cytochrome c oxidase and apocytochrome b) and apicoplast genes (subunit rpoB/C of RNA polymerase), suggesting some activity with these two organelles [34]. A more recent study [35] has shown that doxycycline would specifically act on the apicoplast of *P. falciparum* and, to a lesser extent, on the mitochondrial whose division is inhibited at the end of the cycle; according to the authors, this finding could be attributed to the apicoplastic target (the two organelles present common metabolic pathways). The most recently published study confirms the action of doxycycline on the apicoplast in two stages, with an immediate toxic effect and a toxic effect (measurable after cell division): the first effect is considered to be non-specific, acting on collateral targets that are not located in the apicoplast; the second effect is characteristic of cell death, as observed after an offset effect on the apicoplast [36]. A proteomic approach confirmed the specific deregulation of the proteins involved in apicoplast metabolism after doxycycline treatment [37].

## Antiplasmodial activities

### Activity on sporogony

All studies of the antiplasmodial activity of doxycycline have shown that this molecule, at a dose of 100 mg daily, was a schizonticide agent, with a slow-acting duration [1]. The lack of an in vivo effect of tetracyclines on the development of gametocytes, suggested by Ruiz Sanchez [38, 39], was confirmed by a study performed in 1971 with healthy volunteers infected with *P. falciparum* or *P. vivax* and treated using tetracycline or doxycycline [40]. Tetracyclines have no effect on the sporogony in *Anopheles*: they do not reduce the infectivity of mosquitoes infected with gametocyte carriers under treatment [41].

### Activity on hepatic forms

Several in vivo studies performed with simian models (rhesus monkeys and chimpanzees) infected by *Plasmodium cynomolgi bastianellii*, *P. vivax* or *P. cynomolgi ceylonensis* have shown that terramycin, minocycline or demeclocycline also affected their hepatic forms [42–44]. In a murine model, doxycycline also proved to be effective in the hepatic stages of *Plasmodium berghei* and *Plasmodium yoelii yoelii* [45], as the administration of 1.4 mg of doxycycline simultaneously or 3 h after the injection of sporozoites prevented the appearance of a parasitaemia in 100 % of the rodents (n = 10), while the untreated controls became infected.

However, the activity of doxycycline on the liver forms of *P. falciparum* was demonstrated to be partially effective in several studies of the hepatic forms of *P. falciparum* [46, 47]. Of the twelve subjects who received 100 mg of doxycycline per day for 3 days prior to exposure to infected mosquitoes and for the six following days, four developed malaria [46]. Moreover, the regular uptake of doxycycline did not alter the level of antibodies against the pre-erythrocytic stages of *P. falciparum* [48]. The findings of these studies have justified the recommendation of the currently approved doxycycline regimen (i.e., once daily for 4 weeks after returning from an endemic area).

### Activity on erythrocytic forms

According to Geary et al. [49], cyclines are active during the three developmental asexual erythrocytic stages of *P. falciparum*, equivalently. According to Dahl et al. [35], the aged trophozoites and young schizonts were more susceptible to doxycycline than the young trophozoites and older schizonts, with a dose and time-dependent relationship observed for the effectiveness of the doxycycline on erythrocytic stages. The effectiveness of doxycycline on the erythrocytic stages is evaluated by identifying the concentration necessary to inhibit the growth of 50 % of the parasites, or the IC_50_ [50, 51]. When comparing the IC_50_ value of doxycycline to the values of other anti-malarial drugs, which are sub-micromolar, doxycycline appears to be much less active. Considering its delayed onset of action [52, 53], this finding justifies its therapeutic use in combination with a fast schizonticide.

## Clinical effectiveness

Among tetracyclines, doxycycline is the only one recommended as an anti-malarial prophylaxis [41]. In 1994, 34 years after its development, doxycycline was approved as prophylaxis against malaria by the Food and Drug Administration. In multidrug resistance zones, doxycycline is used as malaria chemoprophylaxis against *P. falciparum* at a dose of 100 mg/day starting at the day of arrival in endemic areas and continuing for up to 4 weeks after returning. This scheme was originally recommended by the WHO in 1985, based on the previously mentioned studies [40, 41]. The primary studies (Table 1) of the efficacy and safety of doxycycline prophylaxis were performed with different populations living in endemic areas [54–58] and non-immune travellers, primarily soldiers from different armies [15, 47, 59]. Most of the failures observed in the prophylaxis of falciparum malaria were related either to inadequate dosages (confirmed by low plasma concentrations of doxycycline) [60], the use of half-doses [55] or poor adherence [59, 61–63]. True prophylactic failures (verified by plasma dosage of doxycycline) are rarely reported. Two Australian soldiers presented with falciparum malaria 2 weeks after returning from Papua New Guinea, despite good adherence [59]. *In vitro* chemosensitivity tests to doxycycline were not performed in these cases. However, the prophylaxis was stopped 3 days after returning from the endemic area; the recommendation is that prophylaxis should be continued 4 weeks after returning. There has been one recent report of the death of a French soldier due to a prophylaxis failure caused by doxycycline resistance [64]. Cyclines are inactive on hypnozoites. Indeed, the occurrence of malaria caused by *P. vivax* or *P. ovale* returning from an endemic area requires a radical cure with primaquine [65].

**Table 1:** Efficacy of doxycycline for prophylaxis against *P. falciparum* malaria

Year	Place	References	Pop	Number	Drug	Route	Dose/d	Other drug	Duration/d	Efficacity
1987	Thailand	Pang [54]	C	95	D	PO	50 or 100 mg^a^	/	35	94.7
1988	Thailand	Pang [55]	C	67	D	PO	50 or 100 mg^a^	/	97	97.0
1988	Thailand	Pang [55]	C	77	D	PO	25 or 50 mg^a^	/	107	97.4
1989	Thailand	Watanasook [56]	A	243	D	PO	50 mg	/	119	92.6
1989	Thailand	Watanasook [56]	A	243	D	PO	100 mg	/	119	84.4
1992	Thailand	Shanks [57]	A	77	D	PO	100 mg	/	80	96.1
1993	New Guinea	Rieckmann [47]	A	60	D	PO	100 mg	/	42	100
1993	New Guinea	Rieckmann [47]	A	69	D	PO	100 mg	PR	21	100
1993	New Guinea	Rieckmann [47]	A	125	D	PO	50 mg	CQ	91	100
1995	Kenya	Weiss [60]	C	32	D	PO	50 mg	/	77	84
1995	Somalia	Shanks [63]	A	900	D	PO	100 mg	/	135	99.9
1995	Cambodia	Shanks [63]	A	600	D	PO	100 mg	CQ	195	99.7
1995	New Guinea	Shanks [59]	A	53	D	PO	100 mg	PR	42	96.2
1997	Irian Jaya	Ohrt [103]	A	67	D	PO	100 mg	/	87	99
1998	Kenya	Andersen [104]	A	70	D	PO	100 mg	/	70	92.6
1999	Irian Jaya	Taylor [58]	A	75	D	PO	100 mg	/	140	96.3
1999	Gabon +CAR	Baudon [15]	A	171	D	PO	100 mg	/	150	97.1
1999	Ethiopia	Schwartz [105]	A	19	D	PO	100 mg	/	/	94.7
2002	Eastern Timor	Peragallo [106]	A	280	D	PO	100 mg	PR	168	98.4
2005	Afghanistan	Sonmez [19]	A	986	D	PO	100 mg	/	84	100

*Pop* population, *A* adults, *C* children, *D* doxycycline, *CQ* chloroquine, *PR* primaquine

^a^According to weight (< or >40 kg)

Doxycycline at a dose of 100 mg/day starting at the day of arrival in endemic areas and continuing for up to 4 weeks after returning, still remains highly effective as *P. falciparum* prophylaxis. Concerning the treatment of malaria, studies conducted in the 1950s [28, 39] and in 1970 [40, 41, 66, 67] have shown the effectiveness of cycline monotherapy in treating simple access to *P. falciparum*. Later, the need for a minimum 7-day treatment was demonstrated; the disappearance of parasites was effective only after 5 days at a dose of 200 mg daily [68].

With the risk of rapid progression from uncomplicated *Plasmodium falciparum* malaria to severe disease and the slow schizonticide action of the cyclines, they should not be used as monotherapy (Table 2). Their combination with other anti-malarial drugs has been studied many times, particularly in areas of multidrug resistance, such as Southeast Asia (Table 3) [69–73]. The most described associations are doxycycline (200 mg) with quinine (10 mg/kg/day) for 7 days, which operates with a therapeutic efficacy of 91–100 % in multi-resistant areas, even if the in vitro susceptibility of isolates to quinine is decreasing [74]. All other tested associations are lower or equal in terms of their efficacy, parasite clearance or resolution of fever, and they are often more expensive.

**Table 2:** Clinical trials of doxycycline monotherapy against *P. falciparum* malaria

Study demographic details					Regimen				
Year	Place	References	Population	Nb	Dosage/d	Nb doses/d	Route	Nb days	Efficacy (%)
1971	USA	Clyde [107]	A	4	200 mg	2	PO	4	NR
1971	USA	Clyde [107]	A	9	200 mg	2	PO	7	NR
1981	West Malaysia	Ponnampalam [68]	C	9	4 mg/kg	NR	PO	4	44.4
1981	West Malaysia	Ponnampalam [68]	C	26	4 mg/kg	NR	PO	7	84.6
2001	Indonesia	Taylor [108]	A	20	200 mg	2	PO	7	64.7

*A* adults, *C* children, *NR* not reported

**Table 3:** Clinical trials of cycline plus other drug against *P. falciparum* malaria

Study demographic details					Regimen								Efficacy (%)
Year	Place	References	Pop	Nb	Cycline			Other Drug			Route	Days	
					Dosage/day	Drug	Nb doses/day	Drug	Dosage/day	Nb doses/day			
1972	Thailand	Colwell [109]	A	30	1000 mg	T	4	Q	1920 mg	3	PO	3 + 10	96.7
1973	Thailand	Chin [69]	A	12	NR	T	NR	Q	NR	NR	PO	NR	66.7
1973	Thailand	Chin [69]	A	13	NR	T	NR	QPy	NR	NR	PO	NR	66.7
1973	Thailand	Colwell [70]	A	32	NR	T	NR	Q	NR	NR	PO	NR	84
1983	Thailand	Noeypatimanond [71]	AC	51	NR	T	NR	Am	NR	NR	PO	NR	96
1988	Cambodia	Giboda [110]	A	22	1500 mg	T	3	Q	1500 mg	3	PO	7/10	100
1994	Thailand	Looreesuwan [111]	A	50	1000 mg	T	4	M	1250 mg	2	PO	7/1	94
1994	Thailand	Looreesuwan [111]	A	52	1000 mg	T	4	Q	1800 mg	3	PO	7	98
1994	Thailand	Looreesuwan [112]	A	54	200 mg	D	1	M	1250 mg	2	PO	7/1	96
1994	Thailand	Looreesuwan [112]	A	55	200 mg	D	1	A	100 mg	2	PO	7/2.5	80
1995	Gabon	Metzger [113]	A	35	4 mg/kg	D	2	Q	24 mg/kg	1	PO	3/1.5	91
1996	Thailand	Na-Bangchang [114]	A	30	200 mg	D	2	A	400 mg	2	PO	5/1	53.3
1996	Thailand	Looreesuwan [115]	A	25	1000 mg	T	4	At	2250 mg	3	PO	7/1.3	100
1996	Thailand	Looreesuwan [115]	A	22	200 mg	D	2	A	1000 mg	2	PO	3	91
1996	Brazil	Duarte [116]	AC	88	1500 mg	T	3	A	100 mg	2	PO	7	80
1996	Brazil	Duarte [116]	AC	88	1500 mg	T	3	Q	2000 mg	2	PO	7/3	77
1996	Thailand	Bunnag [117]	A	46	1000 mg	T	4	Q	1800 mg	3	PO	5	87
1996	Thailand	Bunnag [117]	A	40	1000 mg	T	4	Q	1800 mg	3	PO	7	100
2000	Thailand	Pukrittayakamee [118]	AC	68	16 mg/kg	T	4	Q	30 mg/kg	3	PO	7	98
2001	Indonesia	Taylor [108]	A	39	200 mg	D	2	CQ	STD	1	PO	7/3	90.9
2004	Thailand	Pukrittayakamee [72]	A	30	16 mg/kg	T	4	Q	30 mg/kg	3	PO	7	100
2006	Brazil	Alecrim [74]	A	31	200 mg	D	2	Q	1500 mg	3	PO	3 + 2	100
2007	Pakistan	Ejaz [119]	A	100	200 mg	D	2	Q	30 mg/kg	3	PO	3 + 4	100

*Pop* population, *A* adult, *C* children, *P* pregnant women, *A* artesunate, *Am* amodiaquine, *At* atovaquone, *C* chloroquine, *D* doxycycline, *M* mefloquine, *Q* quinine, *Py* pyrimethamine, *T* tetracycline, *STD* standard, i.e. 10 mg/kg on day 0 and 1 and 5 mg/kg on day 2, *NR* not reported

Due to its slow schizonticide action and short half-life, doxycycline should not be use in monotherapy in the treatment of uncomplicated malaria. Doxycycline remains still effective in combination with quinine or artesunate at a dose of 200 mg for 7 days.

## Mechanism of resistance to doxycycline

The notion of *P. falciparum* resistance to doxycycline is a tricky concept to grasp. Treatment failures reported with quinine plus doxycycline are rare events. The only drug pressure with cycline on *Plasmodium* was performed in a murine model of *Plasmodium berghei* [75]. The administration of increasing doses of minocycline to mice infected with 1 × 10^7^ parasites for 86 successive passages over 600 days made it possible to obtain a resistant *P. berghei* strain, with a median drug inhibitory concentration (IC_50_) of 600 mg/kg/day, which is sixfold higher than that of the susceptible starting strain (100 mg/kg/day).

In addition, few studies have evaluated the *P. falciparum* in vitro susceptibility to doxycycline. However, several studies of isolates from different continents have established different groups of in vitro susceptibility based on IC_50_ doxycycline assessments. But, in the absence of standardized ex vivo and in vitro tests, it is difficult to compare data from different laboratories. Indeed, IC_50_ values and cut-off for in vitro resistance are specific to the methodology. For example, the in vitro effects and the IC_50_ values for doxycycline are dependent upon the time incubation conditions [52, 53], gas conditions (i.e., O_2_ and CO_2_ [76, 77] and methodology (i.e., an isotopic test versus an immunoenzymatic test) [78]. These differences in methodology must be taken account for comparing and analysing resistance data from different works.

A 2010 publication, with reported values of doxycycline IC_50_ on 747 isolates of *P*. *falciparum* in Africa over a period of 9 years (1996–2005), found a trimodal distribution of IC_50_ with three susceptibility levels identified [79]. Nine isolates (1.2 %) exceeded the threshold of 35 µM identifying isolates, with reduced susceptibility to doxycycline. Another evaluation on 484 isolates of imported *P. falciparum* parasites between 2006 and 2010, based on the same methodology, showed that 2.7 % had reduced susceptibility to doxycycline [80]. In a study published in 2013, on 113 isolates from Senegal, 9 (8.0 %) isolates exhibited IC_50_ over the limit of 35 µM [81]. In 2009–2010 and 2010–2011, 12 and 10.3 % of *P. falciparum* isolates collected in Dakar showed reduced susceptibility to doxycycline in comparable methodology (cut off of 37 µM) [78, 82]. A study in Kenya showed that 15 % of the isolates had an IC_50_ >35 µM [83]. A recent study on 620 Thai isolates found a bimodal distribution [84]. The two groups identified presented with a mean value of 13.15 µM for the group of 591 isolates with low IC_50_ and a mean value of 31.60 µM for the group of high IC_50_, including 29 isolates. Only seven isolates of 620 (1.1 %) had doxycycline IC_50_ values that were superior to 35 µM. In 2008, a study performed in French Guiana investigated the prevalence of isolates with reduced susceptibility to doxycycline and found from 15 to 25 % of the isolates from 1996 to 2001, 51 % in 2002, to 61.5 % in 2003 and to more than 67 % in 2005 [50]. The low threshold of susceptibility of 9.6 µM chosen can explain this high level of in vitro resistance. As the methodology is the same as that subsequently used, the prevalence of reduced susceptibility can be recalculated with a cut off at 35 µM: the prevalences ranged from 0 to 4.8 % (0 % in 1997, 1999, 2000, 2003 and 2004, 1.8 % in 1998, 4.8 % in 2001, 2.2 % in 2002 and 1.9 % in 2005). A Ghanaian study performed in 2012 recorded a surprisingly high level of resistance (i.e., 23.7 %) for doxycycline [85], with a threshold IC_50_ value of 35 µM. This finding could be explained by the use of SYBR Green 1-based in vitro test applied to assess the susceptibility of clinical isolates. Indeed, Wein et al. demonstrated that doxycycline IC_50_ values were significantly higher in fluorescence-based SYBR green assays than in isotopic or HRP2-based tests [86]. However, despite the lack of standardization for the evaluation of doxycycline IC_50_, the existence of a high IC_50_ group is indisputable.

The search on the potential mechanisms of the resistance of *P. falciparum* to doxycycline focuses on two ways: the exploration of plasmodial genes homologue to bacterial genes that are involved in bacterial resistance to doxycycline and the exploration of genes coding apicoplastic proteins which could be targets for doxycycline. Different hypotheses have been published regarding the potential mechanisms of the resistance of *P. falciparum* to doxycycline correlated to the bacterial world. Several mechanisms of bacterial resistance to the cyclines have been identified [21]: (1) *tet* efflux protein genes encode for membrane-associated proteins that export tetracycline from the cell, reducing the intracellular drug concentration and thus protecting the ribosomes [87]; (2) TetX protein, a flavin-dependent monooxygenase, degrades tetracycline in vitro and in vivo [88]; and (3) ribosomal protection proteins in the cytoplasm protect ribosomes from the action of tetracycline in a GTP-dependent manner [89, 90]. Analogues of these proteins have been identified in *P. falciparum* [91]. Sequence analysis of 11 genes (*pftufA*, *pfEF*-*TS*, *pfmdt*, *pftetQ*, *pfrps3*, *pfrps7*, *pfrps8*, *pfrps9*, *pfrps11*, *pfrps14*, and *pfrps17*) and evaluation of *pfmdt* and *pftetQ* copy numbers were conducted using 90 isolates from 14 African countries [51]. It has been demonstrated that no polymorphism was found in a small subunit of apicoplastic ribosomal genes (*pfrps7*, *pfrps9*, and *pfrps17*, although S7, S9, and S17) and that the copy number increases of two genes, *P. falciparum* metabolite drug transporter gene (*pfmdt*, PFE0825w), a membrane transporter with similarities to the bacterial efflux pumps, and *P. falciparum* GTPase TetQ gene (*pfTetQ*, PFL1710c), similar to the bacterial ribosomal protein TetA involved in tetracycline resistance, were associated with reduced susceptibility to doxycycline in *P. falciparum* [51]. The number of parasites that is classed as in vitro resistant is very small, and unfortunately, that means that small random changes may be associated without being causal. However, this association was later confirmed using 89 African imported isolates [80]. In addition, PfTetQ KYNNNN motif repeats of <3 are predictive of in vitro resistant *P. falciparum* parasites with IC_50_ >35 µM (odds ratio 15) [83]. The involvement of the copy numbers of *pfmdt* and the PfTetQ KYNNNN motif repeats in reduced susceptibility to doxycycline was confirmed by the doxycycline prophylactic failure from the Central African Republic (i.e., the doxycycline failure in a compliant patient, as confirmed by a statement of correct intake of doxycycline and the presence of an expected plasmatic concentration of doxycycline), which was associated with two copies of the *Pfmdt* gene, as well as the two KYNNNN motif repeats [64]. However, these molecular markers were certainly not the only involved in cases of reduced susceptibility to doxycycline. A study of Senegalese isolates showed a lack of association between the number of copies of *pfmdt* and *pftetQ* and high IC_50_ for doxycycline, essentially because of an insufficient number of isolates with high IC_50_ [81]. There was an absence of association between the number of copies of *pfmdt* and *pftetQ* or the polymorphisms on *pftetQ* and susceptibility to doxycycline in *P. falciparum* isolates from Thailand and French Guiana [84, 92]. Copy number of *pfmdt* and *pftetQ* and polymorphisms on *pftetQ* are not sufficient to explain reduced susceptibility to doxycycline, which may be multigenic.

Other hypotheses were explored. Through homology with the bacterial world, the exploration of new apicoplast genes has been performed, and in particular, the association between the polymorphism of the small subunit ribosomal RNA gene, *pfssrRNA,* and in vitro susceptibility to doxycycline was investigated [93]. In *Helicobacter pylori*, tetracycline resistance has not been associated with efflux or ribosomal protection proteins; instead, it was attributed to mutations in the 16S rRNA-encoding genes that affect the binding site of tetracycline [94, 95]. Tetracycline resistance mediated by mutations in the 16S rRNA was first found in *Propionibacterium acnes*, and a mutation from G to C was reported at position 1058 (*Escherichia coli* numbering) in their 16S rRNA genes [96]. A triplet mutation in the same 16S rRNA domain (965–967; *E. coli* numbering) was also found [90, 95, 97, 98]. Because the apicoplast contains an independent genome, encoding prokaryote-like RNA polymerase subunits, 70S ribosomal subunits, tRNAs and a small number of proteins [99], it was interesting to investigate the mechanism of bacterial resistance of *P. falciparum* to doxycycline. Moreover, comparative analyses of the *P. falciparum* genome revealed that the nucleic acid sequence of a small subunit of ribosomal RNA gene belonging to the apicoplast shares 58 and 62 % of their identities with the 16S rRNA gene from *Propionibacterium acnes* and *Helicobacter pylori*, respectively. However, the sequencing of the small subunit ribosomal RNA gene (PFC10_API0057) in *P. falciparum* African and Thaï isolates did not reveal any mutation, regardless of the determined IC_50_ values [93].

Another hypothesis to be explored is the role of plasmodial apicoplast genes, that bacterial homologues are not involved in bacterial resistance to doxycycline, such as *arps10*, could be involved in artemisinin resistance [100] by encoding the apicoplast ribosomal protein S10 precursor, as well as *fd*, by encoding the ferredoxin protein, a key component of the apicoplast electron transport chain. These apicoplast genes could also be involved in the decreased susceptibility of *P. falciparum* to doxycycline because of doxycycline mode of action.

However, the better way to identify the potential genes involved in reduced susceptibility to doxycycline is to create in vitro resistant parasites in cultivation by drug pressure and then to sequence and analyse the whole genome of the both original susceptible strain and resistant strain as it was successfully previously done for the artemisinin resistance [101, 102].

## Conclusions

The emergence and rapid extension of *P. falciparum* resistance to principal anti-malarial drugs necessitates the search for new molecules. In addition, doxycycline (in combination with quinine) is an excellent candidate for the treatment of uncomplicated malaria and as prophylaxis in multi-resistant areas. The adequate tolerance and efficacy of cyclines have been demonstrated. A better comprehension of the mechanisms of action and resistance would facilitate the design of more effective structural analogues and the identification of molecular markers of resistance to predict and survey the emergence of resistance.
